# Mini-Gastric Bypass/One-Anastomosis Gastric Bypass—Standardizing the Name

**DOI:** 10.1007/s11695-015-1605-y

**Published:** 2015-02-18

**Authors:** Miguel A. Carbajo, Enrique Luque-de-León

**Affiliations:** 1 Centre of Excellence for the Study and Treatment of Obesity and Diabetes, Valladolid, Spain; 2 Centre of Excellence for the Study and Treatment of Obesity and Diabetes-Latinamerican Affiliated Subsidiary, Mexico City, Mexico; 3 The American British Cowdray Medical Center, I.A.P., Mexico City, Mexico; 4 Hospital de Especialidades-Centro Médico Nacional Siglo XXI, Mexico City, Mexico

To the Editor:

After performing many of the alternatives in bariatric surgery during more than two decades, we read with interest the initial ideas Dr. Rutledge proposed in regard to the “mini-gastric bypass” (MGB) and embraced the possibility of performing a very effective operation with fewer risks [1]. Concerned about its major criticism, we modified the original version of the MGB in order to counteract the possibility of alkaline reflux and its sequelae by providing an anti-reflux mechanism; since the beginning and through time, other adjustments to the technique were done and have been [2, 3] and will be published elsewhere.

In 2005, we published the results of our original series with over 200 patients [2] and coined the term “one-anastomosis gastric bypass” (OAGB) for this procedure (BAGUA*—Bypass Gástrico de Una Anastomosis,* in Spanish). We were quite positively impressed with the results, and since 2002, we have adopted it as our main procedure for almost all kinds of patients being submitted both to primary and revisional operations. Our series is now of over 2500 patients and we will soon publish the long-term (6 to 12 years) follow-up of our initial 1200 patients which was recently presented at the 2014 IFSO meeting [3].

The paucity of publications in regard to the MGB/OAGB which characterized the last decade has been changing in the last years, and there are now several publications from around the world, of series, comparative studies, randomized controlled trials, and even systematic reviews [4–6]. This has brought about a controversy regarding the name for the procedure [7–9].

Billroth II and omega loop gastric bypass were seldom used by some groups in the past. Regarding the recent proposal by Lee [7] to change the name to single-anastomosis gastric bypass, we agree with everything stated by Deitel et al. [8] and Rutledge [9] in regard to the confusion that would arise, especially with the various single-anastomosis duodenoileal bypass (SADI-S) procedures. The change in name of the IFSO 2014 Montreal Course from “MGB/OAGB” to “SAGB” indeed led to confusion and even made us change the title of our presentations from OAGB to SAGB [3] in order to be congruent with the title of this first postgraduate course on the subject. Deitel et al. are also correct in expressing that BAGUA can really be translated to OAGB or SAGB in English, so why bother?

Although we know it would be almost impossible (and unfair) to abandon the original term (MGB), the main problem we found with it relies on the fact that it “minimizes” the procedure. As an example of this, we have been asked by our colleagues why are we performing “partial” or “incomplete” bypasses, instead of the standard (complete) procedure! Since we believe its main attributes are effectiveness and safety, and not easiness and rapidness, we strongly believe calling it “mini” diminishes the perception of its real power and deviates attention from its more robust characteristics as an excellent alternative in bariatric and metabolic surgery.

We appreciate the recommendation of leaders in the field [8, 9] in considering OAGB as the only standing alternative name for the MGB, in order to reconcile terms and facilitate issues in the editing and publishing of future related courses and publications. We call on the various bariatric teams that are performing the original MGB or our modified version, the OAGB, to aid in the dissemination and acceptance of this procedure by presenting and publishing their experiences and standardizing the name (to MGB/OAGB), in order for all of us to be recognized as a whole.

Now that many of its controversies are being surpassed and the bariatric surgical community is accepting the procedure as a rational alternative in the bariatric repertoire, we should make all efforts in order to conciliate in regard to the name, avoid new disagreements, and work towards making the MGB/OAGB mainstream in obesity and metabolic surgery.
